# Functional analysis of the mismatch repair system in bladder cancer

**DOI:** 10.1054/bjoc.2001.1949

**Published:** 2001-08

**Authors:** T Thykjaer, M Christensen, A B Clark, L R T Hansen, T A Kunkel, T F Ørntoft

**Affiliations:** Department of Clinical Biochemistry, Skejby University Hospital, Aarhus N, 8200, Denmark; Laboratory of Molecular Genetics, National Institutes of Environmental Health Sciences, Research Triangle Park, North Carolina, 27709, USA; Faculty of Health, Institute of Molecular Pathology, University of Copenhagen, Copenhagen, DK, 2100, Denmark

**Keywords:** mismatch repair, bladder, microsatellite, MSH3, MSH6

## Abstract

In bladder cancer the observed microsatellite instability indicates that mismatch repair deficiency could be a frequently involved factor in bladder cancer progression. To investigate this hypothesis we analysed extracts of seven bladder cancer cell lines and, as a novel approach, five clinical cancer samples for mismatch repair activity. We found that one cell line (T24) and three of the clinical samples had a reduced repair capacity, measured to ~20% or less. The T24 cell extract was unable to repair a G-G mismatch and showed reduced repair of a 2-base loop, consistent with diminished function of the MSH2-MSH6 heterodimer. The functional assay was combined with measurement for mutation frequency, microsatellite analysis, sequencing, MTT assay, immunohistochemical analysis and RT-PCR analysis of the mismatch repair genes MSH2, MSH3, MSH6, PMS1, PMS2 and MLH1. A >7-fold relative increase in mutation frequency was observed for T24 compared to a bladder cancer cell line with a fully functional mismatch repair system. Neither microsatellite instability, loss of repair nor mismatch repair gene mutations were detected. However, RT-PCR analysis of mRNA levels did detect changes in the ratio of expression of the *Mut* S and *Mut* L homologues. The T24 cell line had the lowest MSH6 expression level of the cell lines tested. Identical RT-PCR analysis of seventeen clinical samples (normal urothelium, 7; pTa low stage, 5; and pT1-4 high stage, 5) indicated a significant change in the expression ratio between MSH3/MSH6 (*P*< 0.004), MSH2/MSH3 (*P*< 0.012) and PMS2/MLH1 *P*< 0.005, in high stage bladder tumours compared to normal urothelium and low stage tumours. Collectively, the data suggest that imbalanced expression of mismatch repair genes could lead to partial loss of mismatch repair activity that is associated with invasive bladder cancer. © 2001 Cancer Research Campaign http://www.bjcancer.com


*British Journal of Cancer* (2001) **85**(4), 568–575
© 2001 Cancer Research Campaign
doi: 10.1054/ bjoc.2001.1949, available online at http://www.idealibrary.com on IDEAL

http://www.bjcancer.com

### Functional analysis of the mismatch repair system in bladder cancer

**T Thykjaer[1], M Christensen[1], AB Clark[2], LRT Hansen[3], TA Kunkel[2] and TF Ørntoft[1]**

[1]Department of Clinical Biochemistry, Skejby University Hospital, 8200-Aarhus N, Denmark; [2]Laboratory of Molecular Genetics, National Institutes of Environmental Health Sciences, Research Triangle Park, North Carolina 27709 USA; and [3]Institute of Molecular Pathology, Faculty of Health, University of Copenhagen, DK-2100 Copenhagen, Denmark

**Summary** In bladder cancer the observed microsatellite instability indicates that mismatch repair deficiency could be a frequently involved factor in bladder cancer progression. To investigate this hypothesis we analysed extracts of seven bladder cancer cell lines and, as a novel approach, five clinical cancer samples for mismatch repair activity. We found that one cell line (T24) and three of the clinical samples had a reduced repair capacity, measured to ~20% or less. The T24 cell extract was unable to repair a G-G mismatch and showed reduced repair of a 2-base loop, consistent with diminished function of the MSH2-MSH6 heterodimer. The functional assay was combined with measurement for mutation frequency, microsatellite analysis, sequencing, MTT assay, immunohistochemical analysis and RT-PCR analysis of the mismatch repair genes MSH2, MSH3, MSH6, PMS1, PMS2 and MLH1. A >7-fold relative increase in mutation frequency was observed for T24 compared to a bladder cancer cell line with a fully functional mismatch repair system. Neither microsatellite instability, loss of repair nor mismatch repair gene mutations were detected. However, RT-PCR analysis of mRNA levels did detect changes in the ratio of expression of the *Mut* S and *Mut* L homologues. The T24 cell line had the lowest MSH6 expression level of the cell lines tested. Identical RT-PCR analysis of seventeen clinical samples (normal urothelium, 7; pTa low stage, 5; and pT1-4 high stage, 5) indicated a significant change in the expression ratio between MSH3/MSH6 ($P < 0.004$), MSH2/MSH3 ($P < 0.012$) and PMS2/MLH1 $P < 0.005$, in high stage bladder tumours compared to normal urothelium and low stage tumours. Collectively, the data suggest that imbalanced expression of mismatch repair genes could lead to partial loss of mismatch repair activity that is associated with invasive bladder cancer. © 2001 Cancer Research Campaign http://www.bjcancer.com

**Keywords**: mismatch repair; bladder; microsatellite; MSH3; MSH6

Bladder cancer is a common cancer with approximately 50 000 new incidents per year in the United States (Wingo et al, 1999). The most frequent bladder cancer form is transitional cell carcinomas (TCCs), which in most cases occur as benign papillomas, with superficial recurrence in approximately 35% of the cases and progression to an invasive life-threathening cancer in 15%. A characteristic molecular feature of bladder cancer is a varying genomic instability. This is most often observed as loss of heterozygosity (LOH), dominated by LOH of parts of chromosome 9 in the early stages (Cairns et al, 1993; Keen et al, 1994; Ruppert et al, 1993), LOH of 3p21–25 (Presti et al, 1991), 4q33–34 (Polacik et al, 1995), 8p21 (Knowles et al, 1993), 17p11–13 (Presti et al, 1991) and 18q21.3 (Brewster et al, 1994) in high stages, and frequent mutations in p53 and Rb (Baker et al, 1989; Miyamoto et al, 1995; Taylor et al, 1996). High frequency of LOH at the MLH1 and MSH2 loci was previously shown in clinical bladder cancer samples (Christensen et al, 1998). Several studies also report microsatellite instability in TCC's (Christensen et al, 1998; Gonzales-Zulueta et al, 1993; Li et al, 1996; Orlow et al, 1994). Microsatellite instability is frequently observed in tumour cells from patients with hereditary nonpolyposis colorectal cancer (HNPCC), but is also observed in a significant subset of sporadic colon, gastric, ovarian, endometrical and small cell lung cancers (Arzimanoglou et al, 1998; Lothe, 1997). This instability can in most cases be connected to mutations in genes involved in the postreplicative mismatch repair system (for review see Buermeyer et al, 1999; Kolodner and Marsischky, 1999; Yu et al, 1999). Bladder cancer is not part of the tumour spectrum of HNPCC. The observed microsatellite instability in bladder cancer is rarely of the 'ladder' type as seen in HNPCC but more often an occurrence of a few extra bands or band shifts (Christensen et al, 1998). Another inactivation mechanism of the mismatch repair system, observed in a significant percentage of sporadic colon cancers, is promoter hypermethylation of the mismatch repair gene MLH1 (Ghimenti et al, 1999; Toyota et al, 1999).

Several studies have shown that the mispair recognition in human postreplicative mismatch repair is due to two separate heterodimers, the MSH2-MSH6 (*mutS*α) – and the MSH2-MSH3 (*mutS*β) complexes. It seems that the two heterodimers have some overlapping affinity. MSH2-MSH6 recognizes base-base mispairs and small insertion-deletion loops, whereas MSH2-MSH3 recognizes small and larger insertion-deletion loops (Genschel et al, 1998; Umar et al, 1998a or b; Umar et al, 1994). The mispair recognition is followed by binding of the heterodimer complex, MLH1-PMS2 (*mutL*α) to the *mutS* complex (Gu et al, 1998; Habraken et al, 1998). Recent studies show that other heterodimer complexes exist and may play a part in the repair process (Wang et al, 1999).

*Received 2 August 2000*
*Revised 30 April 2001*
*Accepted 15 May 2001*

*Correspondence to:* T Thykjaer

The observed microsatellite instability and the frequent occurence of mutations in p53 and RB in TCCs indicate that mismatch repair deficiency could be involved in the progression of bladder cancer.

To investigate this hypothesis we tested the functional status of the mismatch repair system in extracts from urothelial cancer cell lines and clinical bladder cancer samples using a DNA heteroduplex repair assay (Thomas et al, 1995). The functional analysis was combined with analysis for microsatellite instability, expression of the mismatch repair genes MSH2, MSH3, MSH6, PMS1, PMS2, and MLH1, by RT-PCR, Western blotting analysis and immunohistochemical staining for detection of the MLH1, MSH2 and PMS2 proteins.

### MATERIALS AND METHODS

### Cell lines

The following cell lines were used in this study: TK6 a lymphoblastoid cell line. HCV29, HU609, SW780, RT4, HT1376, J82 and T24, all bladder cancer cell lines. HCT116 and HCT15 both colorectal cancer cell lines. The cell lines SW780, RT4, HT1376, J82, T24, HCT116 and HCT15 were obtained from the American Type Culture Collection (ATCC). HCV29 and HU609 were obtained from the Fibiger Instituttet, Copenhagen, Denmark. TK6 and HCT15 were cultured in RPMI supplemented with 10% fetal bovine serum. T24, HCT116 and RT4 were cultured in McCoy media supplemented with 10% fetal bovine serum. HT1376 and J82 were cultured in MEM with 10% fetal bovine serum. All media were obtained from Life Technologies Inc., USA.

### Tissue samples

Normal bladder tissue was obtained as biopsies from patients with benign prostatic hypoplasia and urinary incontinence. Tissue samples of transitional cell carcinomas were collected fresh from surgery and immediately put on ice. The tissue samples were disintegrated, permanently embedded in icecold isotonic buffer (Thomas et al, 1995), and then moved through a 100 micron filter (PGC Scientific, Maryland, USA) to ensure single cell preparation and to minimize bladder wall contamination.

At this stage an aliquot of cells was stored in guadinium thiocyanate for RNA preparation. The rest of the cells were used to prepare extracts.

### Preparation of cytoplasmic protein extract

The procedure for preparation of cytoplasmic protein extract from cell lines is described in detail in (Thomas et al, 1995). To prepare extract from bladder tissue samples, the filtered cells were washed in 10 ml ice cold isotonic buffer (Thomas et al, 1995) and centrifuged at $500 \times g$ for 5 min at 4˚C. The isotonic buffer was removed and the cells resuspended in 10 ml icecold hypotonic buffer (Thomas et al, 1995) and centrifuged at $500 \times g$ for 5 min at 4˚C. The hypotonic buffer was removed and the cells resuspended in hypotonic buffer at a cell concentration of approximately $10^8$ cells/ml. The cells were incubated on ice for 15 min, then disrupted using a 2 ml Dounce homogenizer. It is important that only approximately 80% of the cells are broken to minimize the amount of histones in the extract. The broken cells were moved to an Eppendorf tube and incubated on ice for 15 min, and centrifuged at $2000 \times g$ for 5 min at 4˚C. The supernatant was moved to a new Eppendorf tube and centrifuged at $12\,000 \times g$ for 5 min at 4˚C. The extract was aliquoted and immediately frozen at –80˚C.

### SV40 T-antigen dependent replication

Approximately 100 μg of crude cell extract was used in the replication assay (Roberts and Kunkel, 1993). The assay was performed using a SV40 Large T-antigen Replication Assay Kit (CHIMERx, Milwaukee WI, USA) according to the manufacturer’s instructions.

### Heteroduplex repair assay

The assay is described in detail in Thomas et al (1995), and measures the strand-specific repair of mismatches in M13mp2 DNA. DNA substrates used contain a nick in the minus strand to direct the mismatch repair to this strand and different types of mismatches in the *lacZ*α-complementation reporter gene. Introduction of an unrepaired mismatch substrate into an *E. coli* strain defective in mismatch repair (*mutS* strain) yields blue, white and mixed blue/white M13 plaques in a specific reproducible ratio. Mismatch repair activity in the extract to be tested is detected by a reduced percentage of mixed colour plaques and an increase in the percentage of plaques having the colour encoded by the minus strand relative to the unrepeared control. Mismatch repair efficiency is calculated in percentage as 100 × (1 – [% mixed plaques in the experimental sample / % mixed plaques in the mock treated sample]). Repair values between 0 and 10% represent experimental fluctuation in this assay.

### Microsatellite analysis

For microsatellite analysis of cell lines, single cell clones were obtained by increasing dilution into 96-well microtitre plates. When nearly confluent, individual clones were subcultured in 75 ml flasks for ~ 15 generations before harvesting. Approximately 50 ng of DNA from each pool were used in PCR reactions using HEX or FAM end-labelled primers. The PCR products was analysed on an ABI 377 sequence analyser using Gene Scan software version 2.1. The following microsatellite loci were analysed: Mononucleotide repeat: BAT26. Dinucleotide repeat: D2S119, D5S404, D8S255, D17S261. Tetranucleotide repeat: RB1.

### Western blotting

The cell extracts used for Western blotting were similar to the extract used in the mismatch repair assays. Ten μg crude cell extract was reduced by adding 0.5 M DTT, denatured at 70˚C for 10 min and applied to a 10% polyacrylamide gel (NuPage, Bis-Tris Gel, Novex).

Ten μl diluted protein marker (ECL protein molecular marker, Amersham) were used. The samples were electrophoresed at 200 V for 50 min in an X-CELL system (Novex). Then the proteins were transferred to a PVDF membrane at 30 V for 1 hour followed by blocking for 1 h. The membrane was subsequently washed in 3 × 10 min in PBS buffer pH 7.4 + 0.1% Tween 20. The membrane was incubated with antibodies against MLH1, MSH2 or PMS2 overnight at 4˚C. The membrane was then washed 3 × 10 min in

© 2001 Cancer Research Campaign

PBS buffer pH 7.4 + 0.1% Tween 20, followed by incubation for 1 h with a biotinylated streptavidin horseradish peroxidase complex. The detection reagent (ECL + Western blotting detection system, Amersham) was applied for 5 min. Finally, the membrane was wrapped in plastic, sealed, and scanned in a Phosphorimager, STORM 840 (Molecular Dynamics, Amersham Pharmacia, Sweden).

### Immunohistochemistry

Immunoperoxidase staining for MLH1, MSH2 and PMS2 in formalin-fixed, paraffin embedded tissue sections were performed by a labelled biotin-streptavidin method using 3-amino-ethylcarbazole as chromogen. Four µm tissue sections were mounted on Superfrost/Plus slides. The mounted tissue sections were deparaffinized by incubating the slides at 80˚C for 10 min, immersed in heated oil, 60˚C (Estisol 312, Estichem A/S, Denmark) and rehydrated through graded alcohols (99.9%, 97% and 70%) to water. Antigen retrieval was performed using a microwave TEG method by soaking the slides in TEG buffer, 10 mM TRIS, 0.5 mM EGTA pH 9.0. Then the slides were cooked for 12 min and cooled in the TEG buffer for 15 min. To ensure blocking of endogeneous peroxidase activity the slides were soaked in 1% $H_2O_2$ for 20 min, followed by three rinses in tap water. The slides then were soaked in PBS buffer, 10 mM $PO^{-4}$, 0.145 mM NaCl, pH 7.4 for 2 min. From this point and to the development of the sections the descriptions given by the protocol in the Mouse Immunohistochemistry Detection System, XHCO1, Oncogene Science, Inc, UniTect™, Uniondale, NY were followed. Briefly, non-specific protein binding was blocked by incubating the sections with drops of normal horse serum for 20 min at room temperature (RT). Then the slides were washed in PBS. Tissue sections were incubated overnight at 4˚C with mouse monoclonal antibodies against the MLH1 antibody (clone G168–15, 10 µg/ml, PharMingen, San Diego, CA), against the carboxyl terminal region of the MSH2 antibody, (Ab-2, 3 µg/ml) or against the PMS2 antibody (Ab-1, 7.5 µg/ml). MSH2 and PMS2 were obtained from Oncogene Research, Cambridge, MA. Before use, antibodies were diluted in 1% BSA/PBS. After rinsing three times in PBS, the slides were incubated with biotinylated secondary IgG for 30 min at RT, followed by three rinses in PBS. Then sections were incubated with the ABC reagent, (avidin and biotinlylated horseradish peroxidase) for 30 min at RT, followed by a short rinse in PBS. The slides were then washed in running tap water for 5 min and developed in acetate buffer, 200 mM acetate, pH 5.0 containing 3-amino-ethylcarbazole and 2.5 N, N dimethylformamide for 10 min. Shortly before use, 35% $H_2O_2$ was added. After developing, the slides were washed in running tap water for 5 min. For counter-staining Mayers haematoxylin was used.

### RT-PCR

One µg total RNA was used as starting material for first strand cDNA synthesis. The synthesis was performed using the 'First strand cDNA synthesis kit' from Pharmacia Biotech and 0.5 µg of random primers. The length of the amplicons were: β-actin 621 bp; hMSH3 439 bp; hPMS2 359 bp; hMSH6 288 bp; hMSH2 225 bp; hMLH1 215 bp; and hPMS1 174 bp. Primer sequences can be obtained from the authors. One µl of the 20 µl cDNA reaction mix was used in a 25 µl PCR reaction. The PCR profile was one round at 94˚C for 2 min followed by 29 rounds of 30s at 94˚C, 30 s at 62˚C and 45 s at 72˚C, followed by one final extension for 10 min at 72˚C. Five µl of the RT-PCR products were separated on a ethidium bromide stained 2% agarose gel. The gel images were saved as digitized images using the Eagle Eye II Still Video System (Stratagene, California, USA). The areas of the bands were calculated by The QGEL software version 1.72 (Kendrick Labs Inc., Madison WI, USA) and normalized to β-actin. For all samples to be tested, two independent RT-PCR reactions were performed to ensure reproducibility.

### Determination of the mutation frequency at the $Na^+/K^+$ ATPase locus

The mutation frequencies at the $Na^+/K^+$ ATPase locus were determined by plating HT1376, T24 and HCT15 cells in 1 µM ouabain (Sigma). For HT1376 and HCT15 3 $\times 10^6$ cells were plated, and for T24 $6\times 10^6$ were plated. Cells were incubated for 2 weeks and ouabain resistant colonies were visualized by staining with 0.5% crystal violet in 50% methanol. Colonies of more than 50 cells were counted (Umar et al, 1997).

### MTT assay

The sensitivity to 1-methyl-3-nitro-1-nitrosoguanidine (MNNG, Aldrich) was analysed by a MTT proliferation assay (Roche) in two independent experiments in the MMR deficient bladder cancer line T24 and compared to the MMR proficient bladder cancer line HT1376 and the MMR deficient colon cancer cell line HCT15. Cells were plated in 96-well plates at a density at $5 \times 10^3$ cells/well and allowed to attach O/N. Prior to MNNG treatment cells were treated for 1 h with 25 µM $O^6$-benzylguanine (Sigma) in serum free media. MNNG treatment was carried out at different doses (0–200 µM) in serum free medium for 1 h. After treatment serum was added to a final concentration of 10% and cells were incubated for 48 h. Then 20 µl (5 mg/ml) MTT (3-(4,5-Dimethylthiazol-2-yl)-2,5-diphenyltetrazolium bromide, Sigma) in PBS was added to each well and then incubated for four hours. After this 100 µl 10% SDS/0.01 M HCL solution was added and incubated O/N. Plates were read with absorbance at a wavelength of 570 nm and the background absorbance at 690 nm.

### RESULTS

### Mismatch repair in extracts from urothelial cell lines

The seven bladder cancer cell lines selected for this study showed varying degree of genomic instability ranging from nearly diploid, to severe aneuploidy (Table 1).

The lymphoblastoid cell line TK6, previously shown to be mismatch repair proficient for both single base mismatches and from one to sixteen base loops (Umar et al, 1994) was used as positive control. All cell extracts used in the mismatch repair assays were shown to be competent for SV40 T-antigen dependent replication. The replication test was used to ensure that the extract contained functional protein. As substrate for the in vitro mismatch repair assay, we used M13mp2 substrates containing either a G-G mismatch or a 2 base insertion-deletion loop in the *lacZ* reporter gene, and a nick in the (–) strand 3′ to the mismatch, to direct the mismatch repair reaction to the (–) strand. In both cases, the (+) strand of the DNA encodes a colourless plaque phenotype and the (–) strand encodes a blue plaque phenotype.

 © 2001 Cancer Research Campaign

**Table 1**

| Cell line | Karyotype | Reference |
|---|---|---|
| HCV29 | Modal chromosome number = 49; XY, +der(2)t(2;5)(p?12;q?13), +3, del(4)(p?14), -5, -8, del(11)(p11.2;p14), add(11)(p?15), del(12) (p11.1p12.1), add(14)(q?31), –17, +i(17q), add(20)(q13), +20. | Ørntoft et al, 1996 |
| HU609 | Modal chromosome number = 57; XY, del(1)(p25), +der(1)t(1;?)(q?22;?), del(2)(q31), +der(5)t(3;5)(p13;q32), +6, del(7)(p11.2;p14), +7, –9, +10, +13, +14, +20, +add(20)(q13), +der(20)t(3;20)(p?12.2;q13). | Ørntoft et al, 1996 |
| SW780 | Modal chromosome number = 80 to 90 | Kyriazis et al, 1984 |
| RT4 | (P174) hyperdiploid and hypotetraploid to hypertetraploid with abnormalities including dicentrics, breaks, translocations and minutes | Rigby et al, 1970 |
| HT1376 | Modal chromosome number = 104 to 121 | Rasheed et al, 1977 |
| J82 | (P48) triploid with some tetraploid and hexaploid metaphases. Modal number = 72 including minute marker chromosomes | O'Toole et al, 1978 |
| T24 | Hypodiploidy to hypopentaploidy; stemline 86; 2 to 4 telocentrics; 3 to 4 minutes, hypotetraploid to hypertetraploid with abnormalities including dicentrics, breaks, pulverization, minutes and telocentric markers | Fogh, 1978 |

Extracts of cell lines HCV29, HU609, SW780, RT4 and HT1376 all showed efficient strand-specific repair of both the G-G and 2 loop mismatch as indicated by a reduction in the mixed plaque phenotype (Figure 1A) and a reduced blue/white ratio (not shown) compared to mock treated control. The cell line J82 showed slightly reduced repair of a 2-base loop (30%) (Figure 1A). In case of the cell line T24, we found a more pronounced reduction in mismatch repair capacity, G-G repair was reduced to 6% and 2 base loop repair was reduced to 23% (Figure 1A). The reduced repair activity of T24 was not due to a *trans* acting inhibitory factor, because extract from T24 could not inhibit repair of a G-G mismatch when mixed with extract from the repair proficient cell line TK6 (data not shown).

It is previously shown that the MSH6$^{-}$ cell line HCT15, is deficient in repair of single base mismatches and one base loops, but proficient for repair of larger loops (Drummond et al, 1995; Lettieri et al, 1999). In a similar manner, the mismatch repair capacity of the T24 extract is suggestive of MSH6 deficiency. We therefore sequenced the coding region of the MSH6 gene in T24. No mutations were observed in MSH6. We then also sequenced the coding regions of the mismatch repair genes MLH1 and MSH2. Again, no mutations were detected (data not shown).

### T24 is MNNG sensitive

In two independent experiments no MNNG resistance could be detected in the MMR deficient bladder cancer cell line T24 when compared to the MMR proficient HT1376 (Figure 2). This indicates functional MSH6 and/or MSH2 (Umar et al, 1997) as well as MLH1 (Aebi et al, 1997).

### Increased mutation frequency for T24

To support the observed reduced MMR capasity for T24, we tried to measure the mutation frequency at the *HPRT* locus. Unfortunately, our attempts were unsuccessful, no *HPRT* mutant clones were recovered in $5 \times 10^6$ cells. This indicates more than one X chromosome expressing the *HPRT* gene.

Instead we were able to measure mutation frequencies in the $Na^+/K^+$ ATPase locus. Mutations in this locus result in resistance to ouabain. T24 showed an increased mutation frequency of >7.3-fold relative to the MMR proficient cell line HT1376. The MSH6 deficient colorectal cancer cell line HCT15 had an increase in mutation frequency relative to HT1376 of 53-fold (Table 2).

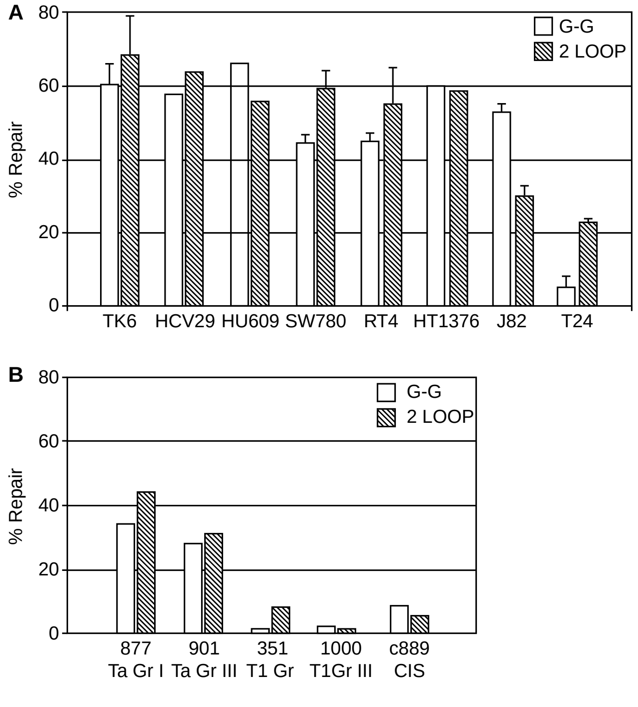

**Figure 1** Mismatch repair measured in crude cell extracts from bladder cancer cell lines and fresh tissue samples from transitional cell carcinomas. The G-G mismatch and 2 base insertion/deletion loop substrates all contained a single strand nick 3′ to the heteroduplex to direct the repair reaction. **A**, mismatch repair efficiency of seven bladder cancer cell lines and as positive control the lymphoblastoid cell line TK6. In most cases the measured repair efficiency is an average of two individual experiments as indicated by the error bars. **B**, mismatch repair efficiency in extract from superficial pTa, submucosa invasive pT1 transitional cell carcinomas, and one carcinoma in situ sample

© 2001 Cancer Research Campaign

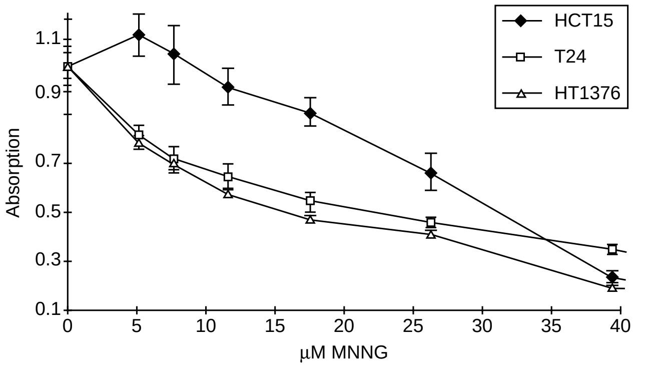

**Figure 2** Examination of MNNG resistance of the cell lines HCT15, HT1376 and T24. Similar results were obtained in two independent experiments. See insets for key to symbols

**Table 2** Mutation frequencies at the $Na^+/K^+$ ATPase locus

| Cell line | Mutation frequency | Relative mutation frequency[a] |
|---|---|---|
| HT1376 | $< 3 \times 10^{-7}$ | 1 |
| T24 | $22 \times 10^{-7}$ | > 7.3 |
| HCT15 | $160 \times 10^{-7}$ | > 53 |

[a]Measured relative to HT1376.

### Microsatellite analysis

In colon cancer, both the hereditary form, known as HNPCC, and the sporadic form, it is known that there is a close correlation between loss of function of the mismatch repair system and microsatellite instability (Fishel et al, 1993; Genuardi et al, 1998; Børresen et al, 1995). We chose four dinucleotide repeats, previously used for microsatellite instability analysis in bladder cancer samples (Christensen et al, 1998), as well as one mononucleotide- and one tetranucleotide repeat. The cell line T24 was tested for microsatellite instability in these six microsatellite loci. None of the tested loci showed instability. The results indicate that the remaining repair capacity in T24 is sufficient to maintain microsatellite stability, although it can not be ruled out that analysis of more microsatellites would reveal unstable loci.

### Western blot analysis

As a supplement to the RT-PCR analysis, the presence of the proteins MSH2, MLH1 and PMS2 were investigated by Western blotting using protein extract from T24 and as positive control TK6. The three proteins were detected in both extracts (Figure 3).

### Mismatch repair in extracts from clinical samples

As a novel approach, we also measured mismatch repair activity in extracts of clinical specimens. We prepared extracts from transitional cell carcinoma samples obtained fresh from surgery. The patient we used for carcinoma in situ material, has been followed for years and had widespread lesions in the bladder. We first tested the extracts' ability to perform SV40 T-antigen dependent replication. Only replication proficient extracts were used in the mismatch repair assay. These extracts were tested for their ability to repair a G-G mismatch and a 2-base insertion-deletion loop. Extracts from the two stage pTa samples showed repair activity, whereas extracts from the two-stage pT1 and the carcinoma in situ sample showed lack of repair activity (Figure 1B).

### Immunohistochemical analysis

Paraffin embedded tissue sections from the clinical samples used in this study were used in immunostaining experiments using antibodies against MSH2, MLH1 and PMS2. Due to the low amount of tissue available from sample 1000 it was not possible to make immunohistochemical analysis on this sample. Strong staining for all three proteins was observed in all the clinical samples tested (Figure 4).

### RT-PCR analysis

Repair of the G-G mismatch used here is known to be initiated by binding of the MSH2-MSH6 heterodimer to the mismatch. The absence of coding sequence mutations in either MSH2 or MSH6 in the T24 cell line suggests that the defect in G-G mismatch repair is due to some other defect. One possibility is imbalanced expression of mismatch repair genes. For example, two studies (Drummond et al, 1997; Marra et al, 1998) have shown that expression of excess

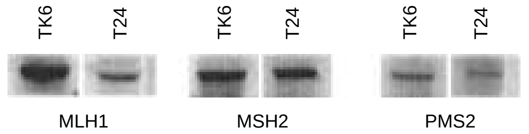

**Figure 3** Western blotting analysis of MLH1, MSH2 and PMS2 in the cell line T24. As positive control the mismatch repair proficient cell line TK6 was used. Each lane contains 10 µg of crude cell extract similar to the extract used in the mismatch repair assays. All three proteins were detected in both cell lines, although the analysis indicate a reduced amount of MLH1 in T24 compared to TK6

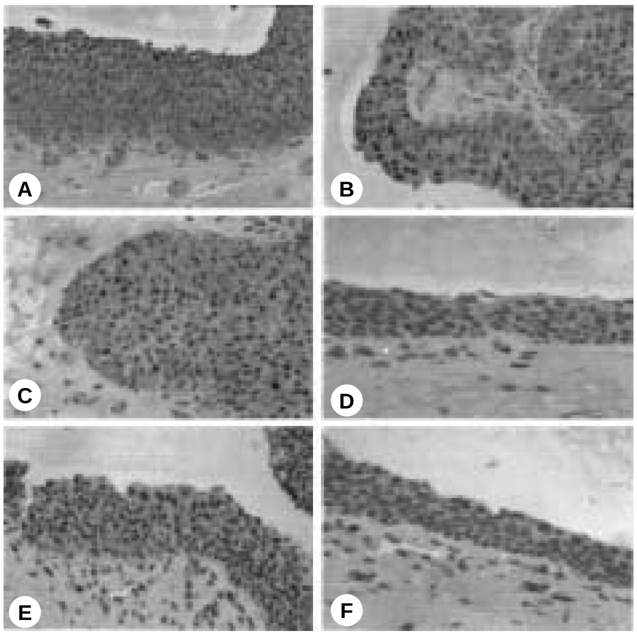

**Figure 4** Examples from the immunohistochemical staining for PMS2, MSH2 and MLH1. 901 is a repair proficient pTa bladder tumour. 351 is repair deficient pT1 tumour. In all cases we could detect positive staining. Sample 901 was stained with antibodies against PMS2 (**A**), MSH2 (**C**) and MLH1 (**E**). Sample 351 was stained with antibodies against PMS2 (**B**), MSH2 (**D**) and MLH1 (**F**)

 © 2001 Cancer Research Campaign

MSH3 leads to reduced base-base mismatch repair capacity. This defect is suggested to result from formation of excess MSH2-MSH3 complex (which does not repair base-base mismatches) with consequent reduction in the level of the MSH2-MSH6 complex that is needed for base-base mismatch repair. Therefore, possible loss of expression or severe changes in expression ratios between the six known mismatch repair genes, MSH2, MSH3, MSH6, PMS1, PMS2 and MLH1 was investigated by RT-PCR (Figure 5A). The analysis was performed on RNA extracted from the seven bladder cancer cell lines used in the mismatch repair assay, two microsatellite unstable colorectal cancer cell lines, seven normal bladder biopsies, five low stage (pTa) and five high stage (pT1-4) bladder cancer tissue samples. The tissue samples 877, 901 and 351 used in the mismatch repair assay were included in the RT-PCR analysis. We could detect PCR products indicating expression of the six mismatch repair genes in all the cell lines and clinical samples investigated (Figure 5). We also tested if we could detect changes in expression ratios between the individual mismatch repair genes. We looked for changes in the expression ratio between MSH3 and MSH6 which compete for binding to MSH2, as well as ratio changes in expression between the two components of the *mutL*α heterodimer complex, PMS2 and MLH1. The PCR reactions were performed by the use of 29 cycles which minimizes the plateau effect of amplification. The intensities of each of the PCR amplicons were analysed by densitometry and normalized to β-actin (Figure 5B). The ratios between the individual amplicons were found to be highly reproducible. The results indicated a general upregulation of the mismatch repair genes in all cell lines and bladder tumor samples compared to normal urothelium. In the bladder cancer cell lines and the two colorectal cancer cell lines, a significant difference from normal urothelium was observed in the PMS2/MLH1 ratio ($P < 0.004$) (Figure 5C). Notably, among the cell lines examined, T24 had the lowest level of expression of MSH2 and MSH6 (arrows in Figure 5B), but a somewhat higher relative level of expression of MSH3. This results in modest changes in the relative ratios of the three *mut*S homologs (arrows in Figure 5C). Also seen was a relative increase in MSH6 and PMS2 expression compared to MSH3 and MLH1 in invasive pT1-4 bladder tumours. The ratios between MSH3/MSH6, MSH2/MSH3, MSH2/MSH6 and PMS2/MLH1 were then calculated (Figure 5C). Using this set-up we could detect a significant reduction in MSH3/MSH6 expression ratio from normal bladder urothelium to high stage tumours ($P < 0.004$) as well as increase in MSH2/MSH3 ratio ($P < 0.012$), and PMS2/MLH1 ratio ($P < 0.005$).

### DISCUSSION

In this study we have investigated the postreplicative mismatch repair system in bladder cancer cell lines and bladder cancer tissue samples. Different methodological approaches were used to analyse the functional status of the mismatch repair system. We combined a functional in vitro assay, measurement of mutation frequencies, microsatellite analysis, Western blotting, immunohistochemistry and RT-PCR in our attempt to reveal possible explanations for the missing or reduced mismatch repair activity we observed in a subset of the samples tested.

One of the seven bladder cancer cell lines, T24, showed significantly reduced repair capacity of a G-G mismatch and, to a lesser extent, reduced repair of a 2-loop substrate. In support of this observation we revealed a >7-fold increase in the mutational

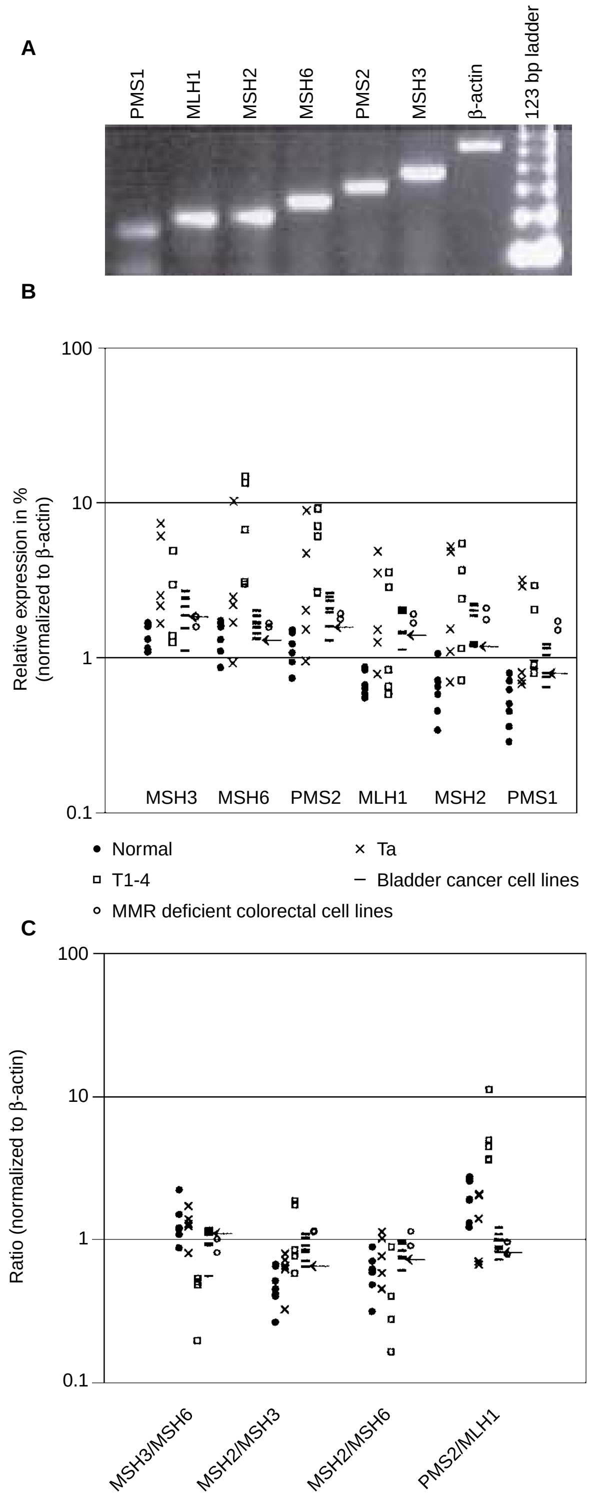

**Figure 5** RT-PCR analysis of the expression of the mismatch repair genes MSH2, MSH3, MSH6, PMS1, PMS2 and MLH1. **A**, shows an example of a gel image used for densiometric analysis. Five μl of the PCR products were loaded on a 2% agarose gel. **B**, Graphic presentation of the densiometric analysis. The figure shows the relative band intensities from seven normal bladder, five pTa, five pT1-4 and seven bladder cancer cell lines, normalized to β-actin. Arrows indicate the measured relative band intensities for the cell line T24. **C**, the ratio between the band intensities of MSH3/MSH6, MSH2/MSH3, MSH2/MSH6 and PMS2/MLH1 for normal urothelium, stage pTa, stage pT1-4 and bladder cancer cell lines were calculated and plotted. Arrows indicate the ratios measured for the cell line T24

© 2001 Cancer Research Campaign frequency at the $Na^+/K^+$ ATPase locus relative to the MMR proficient cell line HT1376. In comparison, the MSH6 deficient colorectal cancer cell line HCT15 showed >53-fold higher mutation frequency compared to HT1376. The RT-PCR analysis of T24 showed the presence of mRNA from all the six known human MMR genes, MSH2-3-6, MLH1, PMS1-2. However, among the seven bladder cancer cell lines examined, T24 had the lowest level of expression of MSH2 and MSH6 and a somewhat higher relative level of expression of MSH3. The resulting modest changes in the relative ratios of the three *mut*S homologs offers one possible explanation for the loss of G-G mismatch repair capasity but retention of microsatellite stability and partial capasity to repair a substrate containing a 2-base insertion/deletion mismatch. This could reflect reduced activity of the MSH2-MSH6 heterodimer (required for repair of base-base but not insertion/deletion mismatches), due to an imbalance in protein levels that allows formation of MSH2-MSH3 complex in excess over MSH2-MSH6 complex. Two previous studies have shown that changes in ratio between MSH3 and MSH6, which both compete for heterodimerization with MSH2, influence mismatch repair activity (Drummond et al, 1997; Marra et al, 1998). Alternatively, the reduced MMR activity might be due a mutation in an unknown MMR gene. The two MMR deficient colorectal cancer cell lines HCT15 and HCT116 showed a relative expression pattern similar to the bladder cancer cell lines. Since T24 cells show severe aneuploidy (Table 1), it is also possible that loss and gain of chromosomal regions could influence the expression level of genes acting further downstream in the repair reaction for example exonucleases, helicases, ligases etc. The MMR phenotype of T24 has similarities to a previously described repair deficient Werner Syndrome fibroblast cell line PSV811 (Bennett et al, 1997), in the way that complementation experiments with a panel of MMR deficient cell lines revealed that PSV811 was not deficient in any of the known MMR genes and furthermore no microsatellite instability was observed.

In this report we also describe functional MMR analysis of five extracts derived from clinical bladder cancer samples obtained fresh from surgery. The composition of the samples was two Ta benign papillomas, two submucosa invasive T1 papillomas, and one sample obtained from a bladder with carcinoma in situ.

The two benign Ta papillomas were repair proficient for both a G-G mismatch and a 2 loop substrate. In contrast the two T1 papillomas and the carcinoma in situ sample showed repair activities less than 10% for both a G-G and two loop substrate. The microsatellite analysis revealed no microsatellite instability in the three repair deficient samples analysing nine microsatellite loci (data not shown). We searched for explanations for the observed lack of, or severely reduced mismatch repair activity in the two T1 and the one carcinoma in situ clinical samples. It was not possible to make further investigations of sample 1000 due to the small amount of tissue available. In the samples 351 and 889 we showed, by immunostaining of tissue sections, that the gene products of MLH1, PMS2 and MSH2 were present. We could measure a significant reduction in the MSH3/MSH6 expression ratio ($P < 0.004$) in high stage tumours compared to normal bladder urothelium, as well as increase in MSH2/MSH3 ratio ($P < 0.012$), and PMS2/MLH1 ratio ($P < 0.005$). The latter change in ratio of *mut*L homologs is also of interest, since a recent report (Shcerbakova and Kunkel, 1999) showed that over-expression of MLH1 in yeast resulted in a mutator phenotype. Further investigations will be required to determine if a change in ratio of the mismatch repair gene products is causing the observed lack of mismatch repair activity in three of the clinical samples. One mechanism leading to altered ratios could be the frequent genomic losses and gains observed in bladder cancer by comparative genomic hybridization analysis (Kallioniemi et al, 1995; Mahdy et al, 1999; Simon et al, 1998; Voorter et al, 1995). A characteristic observation in bladder cancer is a high mutation rate, exemplified by mutations of the TP53 which is mutated in approximately 1/3–1/2 of all tumours. We speculate that a link might exist between the high mutation rate and the observed lack of or reduced repair activity in the three clinical samples and the cell line T24.

### ACKNOWLEDGEMENTS

This work was supported by grants from The Danish Cancer Society, The Karen Elise Jensen Foundation, Aarhus University, and Foundation for the Advancement of Medical Science. We would like to thank Jette Jensen for excellent technical assistance.

### REFERENCES

Aebi S, Fink D, Gordon R, Kim HK, Zheng H, Fink JL and Howell SB (1997) Resistance to cytotoxic drugs in DNA mismatch repair-deficient cells. *Clinical Cancer Research* **3**: 1763–1767

Arzimanoglou II, Gilbert F and Barber HR (1998) Microsatellite instability in human solid tumors. *Cancer* **82**: 1808–1820

Baker SJ, Fearon ER, Nigro JM, Hamilton SR, Preisinger AC, Jessup JM, van Tuinen P, Ledbetter DH, Barker DF and Nakamura Y (1989) Chromosome 17 deletions and p53 gene mutations in colorectal carcinomas. *Science* **244**: 217–221

Bennett SE, Umar A, Oshima J, Monnat RJJ and Kunkel TA (1997) Mismatch repair in extracts of Werner syndrome cell lines. *Cancer Res* **57**: 2956–2960

Børresen AL, Lothe RA, Meling GI, Lystad S, Morrison P, Lipford J, Kane MF, Rognum TO and Kolodner RD (1995) Somatic mutations in the hMSH2 gene in microsatellite unstable colorectal carcinomas. *Hum Mol Genet* **4**: 2065–2072

Brewster SF, Gingell JC, Browne S and Brown KW (1994) Loss of heterozygosity on chromosome 18q is associated with muscle-invasive transitional cell carcinoma of the bladder. *Br J Cancer* **70**: 697–700

Buermeyer AB, Deschenes SM, Baker SM and Liskay RM (1999) Mammalian DNA mismatch repair. *Annu Rev Genet* **33**: 533–564

Cairns P, Shaw ME and Knowles MA (1993) Preliminary mapping of the deleted region of chromosome 9 in bladder cancer. *Cancer Res* **53**: 1230–1232

Christensen M, Jensen MA, Wolf H and Orntoft TF (1998) Pronounced microsatellite instability in transitional cell carcinomas from young patients with bladder cancer. *Int J Cancer* **79**: 396–401

Drummond JT, Li GM, Longley MJ and Modrich P (1995) Isolation of an hMSH2-p160 heterodimer that restores DNA mismatch repair to tumor cells [see comments]. *Science* **268**: 1909–1912

Drummond JT, Genschel J, Wolf E and Modrich P (1997) DHFR/MSH3 amplification in methotrexate-resistant cells alters the hMutSalpha/hMutSbeta ratio and reduces the efficiency of base-base mismatch repair. *Proc Natl Acad Sci USA* **94**: 10144–10149

Fishel R, Lescoe MK, Rao MR, Copeland NG, Jenkins NA, Garber J, Kane M and Kolodner R (1993) The human mutator gene homolog MSH2 and its association with hereditary nonpolyposis colon cancer [published erratum appears in Cell 1994 Apr 8; 77(1):167]. *Cell* **75**: 1027–1038

Fogh J (1978) Cultivation, characterization, and identification of human tumor cells with emphasis on kidney, testis, and bladder tumors. *Natl Cancer Inst Monogr* 5–9

Genschel J, Littman SJ, Drummond JT and Modrich P (1998) Isolation of MutSbeta from human cells and comparison of the mismatch repair specificities of MutSbeta and MutSalpha. *J Biol Chem* **273**: 19895–19901

Genuardi M, Anti M, Capozzi E, Leonardi F, Fornasarig M, Novella E, Bellacosa A, Valenti A, Gasbarrini GB, Roncucci L, Benatti P, Percesepe A, Ponz dL, Coco C, de Paoli A, Valentini M, Boiocchi M, Neri G and Viel A (1998) MLH1 and MSH2 constitutional mutations in colorectal cancer families not meeting the standard criteria for hereditary nonpolyposis colorectal cancer. *Int J Cancer* **75**: 835–839

Ghimenti C, Tannergard P, Wahlberg S, Liu T, Giulianotti PG, Mosca F, Fornaciari G, Bevilacqua G, Lindblom A and Caligo MA (1999) Microsatellite instability

*© 2001 Cancer Research Campaign*

and mismatch repair gene inactivation in sporadic pancreatic and colon tumours. *Br J Cancer* **80**: 11–16

Gonzalez-Zulueta M, Ruppert JM, Tokino K, Tsai YC, Spruck CH, Miyao N, Nichols PW, Hermann GG, Horn T and Steven K (1993) Microsatellite instability in bladder cancer. *Cancer Res* **53**: 5620–5623

Gu L, Hong Y, McCulloch S, Watanabe H and Li GM (1998) ATP-dependent interaction of human mismatch repair proteins and dual role of PCNA in mismatch repair. *Nucleic Acids Res* **26**: 1173–1178

Habraken Y, Sung P, Prakash L and Prakash S (1998) ATP-dependent assembly of a ternary complex consisting of a DNA mismatch and the yeast MSH2-MSH6 and MLH1-PMS1 protein complexes. *J Biol Chem* **273**: 9837–9841

Kallioniemi A, Kallioniemi OP, Citro G, Sauter G, DeVries S, Kerschmann R, Caroll P and Waldman F (1995) Identification of gains and losses of DNA sequences in primary bladder cancer by comparative genomic hybridization. *Genes Chromosomes Cancer* **12**: 213–219

Keen AJ and Knowles MA (1994) Definition of two regions of deletion on chromosome 9 in carcinoma of the bladder. *Oncogene* **9**: 2083–2088

Knowles MA, Shaw ME and Proctor AJ (1993) Deletion mapping of chromosome 8 in cancers of the urinary bladder using restriction fragment length polymorphisms and microsatellite polymorphisms. *Oncogene* **8**: 1357–1364

Kolodner RD and Marsischky GT (1999) Eukaryotic DNA mismatch repair. *Curr Opin Genet Dev* **9**: 89–96

Kyriazis AA, Kyriazis AP, McCombs WB and Peterson WDJ (1984) Morphological, biological, and biochemical characteristics of human bladder transitional cell carcinomas grown in tissue culture and in nude mice. *Cancer Res* **44**: 3997–4005

Lettieri T, Marra G, Aquilina G, Bignami M, Crompton NE, Palombo F and Jiricny J (1999) Effect of hMSH6 cDNA expression on the phenotype of mismatch repair-deficient colon cancer cell line HCT15. *Carcinogenesis* **20**: 373–382

Li M, Zhang ZF, Reuter VE and Cordon-Cardo C (1996) Chromosome 3 allelic losses and microsatellite alterations in transitional cell carcinoma of the urinary bladder. *Am J Pathol* **149**: 229–235

Lothe RA (1997) Microsatellite instability in human solid tumors. *Mol Med Today* **3**: 61–68

Mahdy E, Yoshihiro S, Zech L, Wester K, Pan Y, Busch C, Dohner H, Kallioniemi O, Bergerheim U and Malmstrom PU (1999) Comparison of comparative genomic hybridization, fluorescence in situ hybridization and flow cytometry in urinary bladder cancer. *Anticancer Res* **19**: 7–12

Marra G, Iaccarino I, Lettieri T, Roscilli G, Delmastro P and Jiricny J (1998) Mismatch repair deficiency associated with overexpression of the MSH3 gene. *Proc Natl Acad Sci USA* **95**: 8568–8573

Miyamoto H, Shuin T, Torigoe S, Iwasaki Y and Kubota Y (1995) Retinoblastoma gene mutations in primary human bladder cancer. *Br J Cancer* **71**: 831–835

O'Toole C, Price ZH, Ohnuki Y and Unsgaard B (1978) Ultrastructure, karyology and immunology of a cell line originated from a human transitional-cell carcinoma. *Br J Cancer* **38**: 64–76

Orlow I, Lianes P, Lacombe L, Dalbagni G, Reuter VE and Cordon-Cardo C (1994) Chromosome 9 allelic losses and microsatellite alterations in human bladder tumors. *Cancer Res* **54**: 2848–2851

Ørntoft TF, Meldgaard P, Pedersen B and Wolf H (1996) The blood group ABO gene transcript is down-regulated in human bladder tumors and growth-stimulated urothelial cell lines. *Cancer Res* **56**: 1031–1036

Polascik TJ, Cairns P, Chang WY, Schoenberg MP and Sidransky D (1995) Distinct regions of allelic loss on chromosome 4 in human primary bladder carcinoma. *Cancer Res* **55**: 5396–5399

Presti JCJ, Reuter VE, Galan T, Fair WR and Cordon-Cardo C (1991) Molecular genetic alterations in superficial and locally advanced human bladder cancer. *Cancer Res* **51**: 5405–5409

Rasheed S, Gardner MB, Rongey RW, Nelson-Rees WA and Arnstein P (1977) Human bladder carcinoma: characterization of two new tumor cell lines and search for tumor viruses. *J Natl Cancer Inst* **58**: 881–890

Rigby CC and Franks LM (1970) A human tissue culture cell lines from a transitional cell tumour of the urinary bladder: growth, chromosome pattern and ultrastructure. *Br J Cancer* **24**: 746–754

Roberts JD and Kunkel TA (1993) Fidelity of DNA Replication in Human Cells. *Methods Mol Genet* **2**: 295–313

Ruppert JM, Tokino K and Sidransky D (1993) Evidence for two bladder cancer suppressor loci on human chromosome 9. *Cancer Res* **53**: 5093–5095

Shcherbakova PV and Kunkel TA (1999) Mutator phenotypes conferred by MLH1 overexpression and by heterozygosity for mlh1 mutations. *Mol Cell Biol* **19**: 3177–3183

Simon R, Burger H, Brinkschmidt C, Bocker W, Hertle L and Terpe HJ (1998) Chromosomal aberrations associated with invasion in papillary superficial bladder cancer. *J Pathol* **185**: 345–351

Taylor JA, Li Y, He M, Mason T, Mettlin C, Vogler WJ, Maygarden S and Liu E (1996) p53 mutations in bladder tumors from arylamine-exposed workers. *Cancer Res* **56**: 294–298

Thomas DC, Umar A and Kunkel TA (1995) Measurement of heteroduplex repair in human cell extracts. *Methods: A Companion to Methods in Enzymology* **7**: 187–197

Toyota M, Ahuja N, Ohe-Toyota M, Herman JG, Baylin SB and Issa JP (1999) CpG island methylator phenotype in colorectal cancer. *Proc Natl Acad Sci USA* **96**: 8681–8686

Umar A, Boyer JC and Kunkel TA (1994) DNA loop repair by human cell extracts [see comments]. *Science* **266**: 814–816

Umar A, Koi M, Risinger JI, Glaab WE, Tindall KR, Kolodner RD, Boland CR, Barrett JC and Kunkel TA (1998a) Correction of hypermutability, *N*-Methyl-*N*′-nitro-*N*-nitro-*N*-nitrosoguanidine resistance, and defective DNA mismatch repair by introducing chromosome 2 into human tumor cells with mutations in *MSH2* and *MSH6 Cancer Research* **57**: 3949–3955

Umar A, Risinger JI, Glaab WE, Tindall KR, Barrett JC and Kunkel TA (1998b) Functional overlap in mismatch repair by human MSH3 and MSH6. *Genetics* **148**: 1637–1646

Voorter C, Joos S, Bringuier PP, Vallinga M, Poddighe P, Schalken J, du MS, Ramaekers F, Lichter P and Hopman A (1995) Detection of chromosomal imbalances in transitional cell carcinoma of the bladder by comparative genomic hybridization. *Am J Pathol* **146**: 1341–1354

Wang TF, Kleckner N and Hunter N (1999) Functional specificity of MutL homologs in yeast: evidence for three Mlh1-based heterocomplexes with distinct roles during meiosis in recombination and mismatch correction. *Proc Natl Acad Sci USA* **96**: 13914–13919

Wingo PA, Ries LA, Giovino GA, Miller DS, Rosenberg HM, Shopland DR, Thun MJ and Edwards BK (1999) Annual report to the nation on the status of cancer, 1973–1996, with a special section on lung cancer and tobacco smoking. *J Natl Cancer Inst* **91**: 675–690

Yu Z, Chen J, Ford BN, Brackley ME and Glickman BW (1999) Human DNA repair systems: an overview. *Environ Mol Mutagen* **33**: 3–20

© 2001 Cancer Research Campaign
